# A case for implementation of adult pneumococcal vaccine program in Africa: review and expert opinion

**DOI:** 10.11604/pamj.2022.41.51.31849

**Published:** 2022-01-19

**Authors:** Reena Shah, Catherine Gathu, Eric Njenga, Jeremiah Chakaya, Elijah Ogola, Omondi Oyoo, Andrew Odhiambo, Benjamin Wambugu, Charles Feldman

**Affiliations:** 1 Aga Khan University Hospital, Nairobi, Kenya,; 2 Department of Internal Medicine, Kenyatta University, Nairobi, Kenya,; 3 Department of Clinical Medicine and Therapeutics, College of Health Sciences, University of Nairobi, Nairobi, Kenya,; 4 Department of Medicine, Kenyatta National Hospital, Nairobi, Kenya,; 5 Deparment of Internal Medicine, Faculty of Health Sciences, University of the Witwatersrand, Johannesburg, South Africa

**Keywords:** Pneumococcal, vaccine, opinion

## Abstract

Vaccines are considered as a therapeutic area for children; the scientific community focuses mainly on managing chronic disease when it comes to adults. There currently is an increase in the burden of vaccine preventable illnesses in adults. Adult vaccination has been shown to dramatically increase the health and quality of life of older populations. Therefore, adult vaccinations need to be approached as a public health issue, similar to smoking cessation programs, for example. According to the Kenya Non-Communicable Diseases and injuries poverty commission report, 2018. Kenya has a high percentage of disability adjusted life years (DALYs) from communicable diseases at 63%, while non-communicable diseases (NCDs) contribute 30% of the DALYs. Specific to pneumococcal pneumonia (PP) in adults, the Global burden of disease (GBD) study in 2016 found that 2,377,697 people of all ages died from lower respiratory tract infections (LRTI) in 2016. Of these, more people died from Streptococcus pneumonia(SP) than from all other studied respiratory pathogens combined. While the incidence of LRTIs in children under five years old was reducing, partly as a result of well-established vaccination programs in children, the incidence, morbidity and mortality of PP was increasing in older populations. The expert recommendations included the following; i) all individuals 65 years of age and above, and individuals with a predisposing comorbidity regardless of age, should receive the pneumococcal vaccine; ii) several systemic modules can be emulated from the successful childhood vaccines programs onto an adult vaccine program; iii) formulation of an effective vaccine program will require collaboration from the public, the government, healthcare providers, and the media, to create awareness; iv) stakeholders who need to be involved in vaccine policy development and implementation include medical professional associations, nurses, pharmacists, clinical officers, payers (private and public insurances), government, medical learning institutions and faith-based medical organizations.

## Opinion

**Burden of pneumococcal pneumonia in Kenya:** Kenya has a high percentage of disability adjusted life years (DALYs) from communicable diseases at 63%, while non-communicable diseases (NCDs) contribute 30% of the DALYs [1]. The Global Burden of Disease (GBD) study in 2016 found that 2,377,697 people of all ages died from LRTI in 2016. Of these, more people died from SP than by all other studied respiratory pathogens combined [2]. While the incidence in children under five years of age was reducing, the incidence, morbidity and mortality was increasing in older populations. In the United State alone, among adults, invasive pneumococcal disease carries a mortality of approximately 20-30% against an annual incidence of approximately 8700 cases, while non-bacteraemic pneumococcal pneumonia, which is the major burden in the adult population, carries a mortality of approximately 5-7% against an annual incidence of approximately 500,000 cases [2]. Pneumococcal community-acquired pneumonia (CAP) is by far more common in sub-Saharan Africa than in Europe or North America [3]. Lower respiratory infections are still the leading cause of death in low-income countries. A study in Kibra in Nairobi, Kenya and Western Kenya showed that there is a 90% carriage rate of Streptococcus pneumoniae in these populations based on culture from nasopharyngeal swabs [4].

A rise in the aging population in Kenya is increasing the need for pneumococcal adult vaccination in Kenya. Following an episode of pneumococcal pneumonia, the mortality is increased for up to 10 years, compared to the expected 10 year survival of an average 63-year-old American male [5]. The most common risk factors for acquiring CAP and invasive pneumococcal disease (IPD) included age 6 years, Diabetes mellitus, alcohol misuse disorders, chronic obstructive pulmonary disease (COPD), asthma, smoking, cancer, cerebrovascular disease, chronic kidney disease and HIV [6]. These comorbidities and risk factors are on the rise in the Kenyan adult population. Having two or more comorbid conditions drastically increases the incidence of pneumococcal CAP. In addition, in one study, there was a reciprocal relationship between pneumococcal CAP and comorbid conditions, with patients reporting worsening of their comorbid conditions following an episode of pneumococcal CAP [7]. The morbidity and mortality rate of CAP in adults is possibly greatly underestimated in the Kenyan context due to insufficient research and data

**Impact of adult pneumococcal vaccination:** the community-acquired pneumonia immunization trial in adults (CAPiTA) study was a phase 4, randomized, placebo-controlled clinical trial of the 13-valent pneumococcal conjugate vaccine efficacy in prevention of vaccine-serotype pneumococcal community-acquired pneumonia and invasive pneumococcal disease, which yielded the following results: 46% efficacy in prevention of vaccine-type (VT) CAP, and 75% efficacy in prevention of VT IPD [8]. Post hoc analysis of CAPiTA showed significant and persistent efficacy of PCV13 against VT-CAP in at-risk older adults [9]. Furthermore, COPD exacerbations were reduced, as was risk of hospital admission among COPD patients in vaccinated cases [9]. There is high vaccine coverage of children of nearly 100% by pneumococcal vaccines as a result of initiatives from the Ministry of Health, Kenya (MOH), which has reduced the incidence of CAP in children, as evidenced by a demographic health Survey in Kilifi, 2011 whereby IPD sharply declined both in vaccinated and unvaccinated children following introduction of PCV 10 [10].

**Challenges in attaining adequate adult vaccination coverage for pneumococcal pneumonia in Kenya:** vaccination advice for adults is hereditary coproporphyria (HCP) initiated. The majority of the HCPs are under-informed or uninformed of the existence and indications of PP vaccines for adults. The existing policies and protocols from the Ministry of Health are non-specific, not recently updated, and not widely disseminated to the HCPs. There are no local studies illustrating the burden of non-vaccination in adults or the benefits of vaccination. Members of the public are not aware of the huge impact of adult pneumococcal vaccinations, with the belief that vaccines “only exist” for children. In addition, the “anti-vaxxer” movements have gained momentum in causing pensiveness towards vaccines, making uptake of vaccines in general challenging. Adult pneumococcal vaccines are costly, making them inaccessible to a great majority of the population. There are no programs in Kenya that provide finance or reimbursements for adult vaccines (i.e. NHIF/private insurances/government or nongovernmental reimbursement schemes). The vaccines are not freely available in the government-run health facilities, which is where the majority of patients are treated. There is insufficient infrastructure in the country to reliably maintain the cold chain required of vaccines. Poor inventory management leads to regular stock-outs of vaccines in the country. There is no national registry for adult vaccinations in Kenya at the moment.

**Figure 1:**
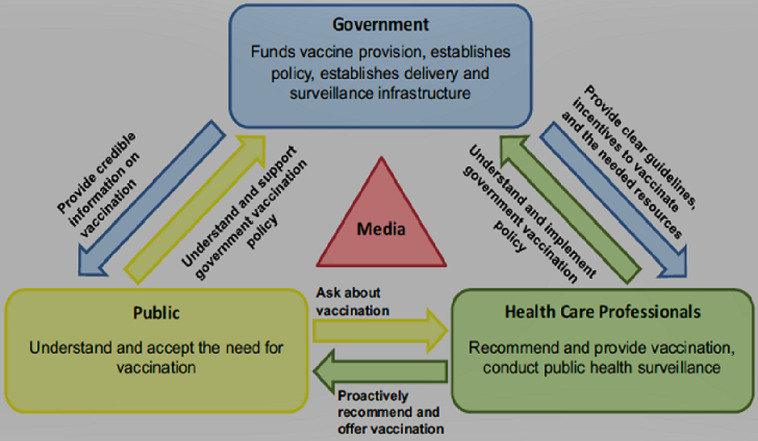
stakeholder involvement in adult vaccine program implementation

**Recommendations/framework for developing and implementing pneumococcal adult vaccine program in Kenya:** all key stakeholders must be involved in the process of developing and implementing the pneumococcal vaccine program in Kenya as illustrated in Figure 1 [11]. Other stakeholders not captured in the Figure 1 are: bodies involved in accessibility of the vaccines (NHIF and private insurers), religious organizations which run several major health facilities christian health association of Kenya (CHAK) and council of catholic bishops and medical learning institutions. Under HCPs, professional associations are to be involved; notably the nursing Council, the clinical officers´ council, pharmacy and poisons board, paediatrician associations, infectious disease experts, family medicine associations, physician associations, pulmonology associations, rheumatology associations, oncology associations and other specialist organizations with specialists that cater to the at-risk populations. With regard to government, both local county and national government representatives should be involved. National government is to be represented by the Ministry of Health, with inclusion of the non-communicable disease department in the Ministry of Health. Local government is to be represented by the association of County Executive Committee Members for Health. The public is to be involved directly, via media campaigns and through patient groups.

Development of the vaccines program could borrow from the already established and successful paediatric vaccination program. Local data should be collected via surveys, research and interventional studies to determine the burden of PP in Kenya, the burden of non-vaccination of PP and the benefit of adult vaccination in Kenya. This data should be shared with the intended financiers and reimbursement bodies to illustrate the need for adult PP vaccination programs in the country. With involvement of the MOH and the named stakeholders, the existing guidelines on pneumococcal vaccination need to be updated, approved and disseminated to all relevant HCPs and the public. HCPs, directly and through the respective associations, need to be trained on the significance and administration of the PP vaccine. Training should be conducted directly to all the aforementioned HCP cadres via seminars, conferences, advisory boards and dissemination of education material on the subject matter. There should be an establishment of an electronic medical register by the ministry of health recording pneumococcal adult vaccinations in Kenya.
